# Rethinking Human Energy Metabolism

**DOI:** 10.3390/cimb48020159

**Published:** 2026-02-01

**Authors:** Alexander Panov, Vladimir Mayorov, Sergey Dikalov, Alexandra Krasilnikova, Lev Yaguzhinsky

**Affiliations:** 1 Department of Biomedical Sciences, School of Medicine, Mercer University, Macon, GA 31201, USA; mayorov_vi@mercer.edu; 2 Vanderbilt University Medical Center, Nashville, TN 37232, USA; sergey.dikalov@vanderbilt.edu; 3 Department for Personalized and Preventive Medicine, Institute for Interdisciplinary Medicine, Burevectnick Business Centre, 18/19 3-rd Rybinskaya Street, Moscow 107113, Russia; alexpro.biochem@gmail.com; 4 Belozersky Institute of Physico-Chemical Biology, Lomonosov Moscow State University, Leninskye Gory 1/40, Moscow 119992, Russia; yag@genebee.msu.ru

**Keywords:** beta-oxidation of fatty acids, energy metabolism, fatty acids, glycolysis, lactate, lactate cycle, mitochondria, pyruvate, respirasome, tricarboxylic acid cycle

## Abstract

For a long time, glycolysis and mitochondrial oxidative phosphorylation were opposed to each other. Glycolysis works when there is a lack of oxygen; the mitochondria supply ATP in an oxygen environment. In recent decades, it has been discovered that glycolysis in vivo always works and the final product is lactate. Lactate can accumulate and is the transport form for pyruvate. In this review, we look at how obligate lactate formation during glycolysis affects the tricarboxylic acid (TCA) cycle and mitochondrial respiration. We conclude that fatty acid β-oxidation is a prerequisite for obligate lactate formation during glycolysis, which in turn promotes and enhances the anaplerotic functions of the TCA cycle. In this way, a supply of two types of substrates for mitochondria is formed: fatty acids as the basic energy substrates, and lactate as an emergency substrate for the heart, skeletal muscles, and brain. High steady-state levels of lactate and ATP, supported by β-oxidation, stimulate gluconeogenesis and thus support the lactate cycle. It is concluded that mitochondrial fatty acids β-oxidation and glycolysis constitute a single interdependent system of energy metabolism of the human body.

## 1. Introduction

Energy metabolism in humans is the complex network of biochemical catabolic, anabolic, and anaplerotic processes that convert food nutrients first into the following usable substrates: glucose, fats, and amino acids, which are then converted into usable energy. The latter, in addition to ATP, also includes NADPH, differences between the cell’s compartments in concentrations of protons, electrical charges, and metabolites. There are two main energy-producing processes: glycolysis and mitochondrial oxidative phosphorylation. For some reasons, which we discuss below, fatty acids β-oxidation was not studied at the mitochondrial level. This resulted in misconceptions that the energy production during metabolism of glucose and lactate is, obviously, independent of fatty acid β-oxidation [1,2,3]. Some authors never mentioned that fatty acids exist as mitochondrial substrates.

New discoveries indicate a very complex structural and functional organization of oxidative phosphorylation enzymes. This makes us reconsider our ideas about humans’ energy metabolism. In this review, we analyze the relationships between the two main metabolic pathways of energy production: glycolysis and mitochondrial oxidative phosphorylation. We came to the conclusion that both mechanisms of ATP production are interdependent, providing high productivity and plasticity of the human energy metabolism.

### Oxidative Phosphorylation

**Figure 1:**
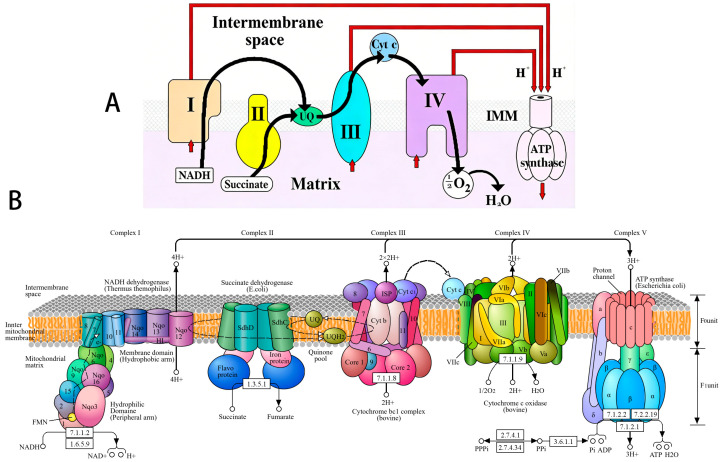
Structure and function of the mitochondrial respiratory chain according to the old paradigm. (**A**) An early scheme of the respiratory chain; (**B**) a more sophisticated presentation of the respiratory chain (this figure was reprinted from [5]).

OXPHOS is the primary function of mitochondria. For decades, numerous mitochondrial functions have been studied in the in vitro system using isolated mitochondria. The respiratory chain of mitochondria in all mammals is basically the same, since it has been an effective energy-producing device from the very beginning of evolution [4]. According to a long tradition, the sequence of protein complexes that are involved in electron transport and ATP synthesis are divided into five functional complexes, which are numbered in order: complexes I, II, III, IV, and V. Such numbering was associated with the logic of events in oxidative phosphorylation that existed at that time (the middle of the 20th century). Complexes I, III, and IV are involved in the transfer of protons and generation of the transmembrane potential (Δp = ΔΨ − 59 ΔpH). Complex V is ATP syntase which consumes ΔpH and ATP/ADP antiporters (adenine nucleotide translocase—ANT) consume ΔΨ. This raises the energetic value (∆G^o^) of ATP from −7.3 kcal/mol to about −14.0 kcal/mol. For a long time, the role of ANT was not taken into account in the overall energy balance of oxidative phosphorylation. Complexes II, also known as succinate dehydrogenases (SDH), do not directly create a ∆pH or ΔΨ gradients, but they are the only enzymes in the tricarboxylic acid cycle (TCA) embedded into the inner membrane of mitochondria. SDH reduces the membrane’s pool of ubiquinone (coenzyme Q) to ubiquinol (coenzyme QH_2_), which is oxidized by the respiratory complex III. The names of the complexes reflect their main function as follows: complex I (CI; NADH:ubiquinone (UQ) oxidoreductase), complex II (CII; succinate:UQ oxidoreductase), complex III (CIII; cytochrome *bc*1), and complex IV (CIV; cytochrome *c* oxidase). Figure 1 reflects how our knowledge about the structure of the respiratory chain changed with time.

Unfortunately, the classical ideas about the structure and function of the respiratory chain turned out to be incorrect. In 2000, it was shown that the respiratory chain of the electron transporters is organized into superstructures called “respirasomes”. Each respirasome consists of three supercomplexes; two of them contain CI, one dimer of CIII, and two dimers of CIV; the third, smaller supercomplex, contains one dimer of CIII and two dimers of CIV [6,7]. Together, the three supercomplexes form the repirasome that also physically interacts with the polyenzymatic structures of the fatty acids β-oxidation of [8]. It was soon discovered that these complex structures of the respirasome form a higher-order structure, a chain [9,10,11,12]. In turn, functionally and structurally, the chain of respirasomes interacts with the chain built from ATP synthase dimers and adenine nucleotide transporters (ANT). The ATP synthase chains are located in the negative curvatures of the cristae membrane, and the respirasome chain is located parallel to the ATP synthase chain at a distance that allows protons to move from respirasomes to ATP synthases without losing energy [11,12].

At first, the physiological significance of the megastructural construction of the respirasome seemed incomprehensible. However, taking into account the known properties of the large supercomplexes containing complex I and the new data upon activation of the reverse electron transfer through SDH from CoQH_2_ to the TCA cycle, it was proposed that the main function of the respirasome is to carry out the β-oxidation of the long-chain and middle-chain fatty acids [13].

The third, smaller supercomplex of the respirasome contains only a dimer of complex III and two dimers of complex IV (CIII_2_CIV_4_) [7]. This supercomplex directly irreversibly oxidizes at a very high rate the inner membrane’s pool of CoQH_2_ (ubiquinol). Due to the presence of the Q cycle in complex III, the rate of ubiquinol oxidation, although very high, is finite [14]. In addition, the rate of oxygen consumption by mitochondria is controlled by the rate of ATP synthases [13]. The highest steady-state levels of the coenzyme Q reduction are observed during fatty acid β-oxidation [15]. The high rate of ubiquinone reduction is achieved by the coordinated work of five mitochondrial acyl-CoA dehydrogenases as follows: there are long-, medium-, and short-chain acyl-CoA dehydrogenases (LCAD, MCAD, and SCAD), which are most active with 14, 8, and 4 carbon substrates, respectively. These acyl-CoA dehydrogenases are homotetramers [16,17]. Very long-chain acetyl-CoA dehydrogenase (VLCAD has optimal chain length specificity for fatty acyl-CoAs with sixteen carbons [18]). The fifth enzyme, human mitochondrial acyl-CoA dehydrogenase-9, plays a role in the mitochondrial β-oxidation of unsaturated fatty acids [19]. In addition, acyl-CoA dehydrogenase-9 is required for the biogenesis of the respiratory complex I [20]. VLCAD and ACAD-9 are dimers and are associated with the inner membrane. Thus, altogether, approximately 14–16 acyl-CoA dehydrogenases of a single β-oxidation set of enzymes may work simultaneously, maintaining the high steady-state level of ubiquinol in spite of its very fast oxidation by the smaller supercomplex.

At the high level of ubiqunol, SDH initiates the reverse electron transport creating high levels of reduction in the cell’s NADH/NAD+ and NADPH/NADP+ systems, as well as ATP production [15]. As a result, in vivo, glycolysis always produces lactate instead of pyruvate. A large body of evidence proves that lactate and the lactate cycle play a crucial role in the overall energy balance in the body [21,22,23]. To grasp and to understand the consequences of the obligatory production of lactate, due to activation of the fatty acids β-oxidation, is the goal of this review.

## 2. A Quick Critical Look at the Energy Metabolism from the Point of View of the New Paradigms

The driving force for the movement of electrons down the respiratory chain is the difference in the redox potential between hydrogen, as the primary source of energy, and water, as the final product. The redox potential is a measure of the readiness of a compound to give or accept electrons. Compounds with lower redox potential are ready to give away electrons to compounds with higher redox potentials that have a higher affinity for electrons.

Normally, electrons are transported through the respiratory chain one at a time. When tracing the path of one electron from the reduced pyridine nucleotide (NADH), the first reaction of NADH will be with the enzyme NADH dehydrogenase, which is a component of the respiratory complex I and contains flavinmononucleotide (FMN). This is the rate-limiting step of the whole pathway of an electron, if mitochondria oxidize NADH only. Electrons from the reduced FMN are transferred one by one along the chain of the Fe-S clusters that form an isolated tunnel for electrons. The electrons are transported onto the complex-I-bound coenzyme Q (CoQ), reducing it to CoQH_2_. The reduced coenzyme CoQH_2_ (ubiquinol) of the complex I reacts directly with the tightly bound CoQ of the complex III, reducing it. Then, electrons are transferred through the channel for electrons to the terminal cytochrome c1. Reduced cytochrome c1 transfers electrons to cytochrome c. Reduced cytochrome c is oxidized by complex IV, where the final reaction of oxygen (O_2_) reduction to 2H_2_O takes place. This final reaction creates a large amount of heat and is the site of irreversibility. In accordance with the old paradigm, this is the sequence of events in the respiratory chain of mitochondria oxidizing the so-called complex I substrates. It was generally accepted that NADH, the actual substrate for the respiratory chain, was supplied by the tricarboxylic acid cycle, which is started by condensation of acetyl-CoA and oxaloacetate (OAA). Most researchers considered pyruvate as the main source of acetyl-CoA for the TCA cycle and pyruvate as the final product of glycolysis, which is wrong.

### 2.1. Glycolysis, Tricarboxylic Acid Cycle, and the Mitochondrial Substrates

In the early decades of mitochondrial research, it was crucial for researchers to achieve a stable and possibly higher energy state of the isolated mitochondria. Researchers used different substrates to energize mitochondria in the experiments. However, from the very beginning they encountered serious problems with the two key substrates, namely succinate and fatty acyl-carnitines. Mitochondria isolated from the brain and heart were unable oxidize succinate alone, but succinate + rotenone was a perfect substrate for mitochondria isolated from any organ. As regards acyl-carnitines, which in vivo are normal respiratory substrates for all types of mitochondria [24], in experiments in vitro the rates of β-oxidation were too slow to be physiologically relevant. For this reason, until recently, we knew very little about β-oxidation of long-chain and middle-chain fatty acids at the mitochondrial level. As a result, most of our knowledge about the energy-dependent functions of mitochondria was obtained on mitochondria energized either with the absolutely non-physiological substrate succinate + rotenone, or other physiologically irrelevant substrates. For example, glutamate, which is a too valuable metabolite to be used as a sole source of energy in vivo, or pyruvate, which by itself is not a substrate for the tricarboxylic acids (TCAs) cycle.

### 2.2. The Tricarboxylic Acid Cycle

As stated above, in vivo, glycolysis does not produce pyruvate as a substrate for the mitochondria [25]. For this reason, in most cases, the TCA cycle begins with acetyl-CoA, which originates from the fatty acids’ β-oxidation. Researchers, however, often used pyruvate + malate as substrates for the isolated mitochondria from most organs, except the liver. This is because in the fasted overnight animals, the liver pyruvate dehydrogenase complex is inhibited. Glutamate + malate was also a popular substrate for all types of mitochondria. Many researchers regarded pyruvate and glutamate as the specific substrates for complex I, whereas succinate (usually with rotenone) served as a substrate for complex II. However, in the mitochondria of most organs, pyruvate, and especially glutamate, are metabolized via transamination to form a-ketoglutarate and then succinate.

For a long time, it was believed that the enzymes of the TCA cycle reside in the matrix of mitochondria and the respiratory chain was presented as a sequence of the respiratory complexes, not as a respirasome. Already in the 1990s, it was known that quantitatively, the TCA cycle enzymes associated with the inner membrane, 200 times exceeded those in the matrix [26]. Now we know that some of the experimental complications observed with the isolated mitochondria stem from the fact that practically all researchers utilized only one substrate or in combination with malate. By itself, malate is not oxidized by the mitochondria, but malate helps to oxidize some other substrates because it is the source of oxaloacetate. In vivo, mitochondria always oxidize mixtures of substrates that are optimal for a given organ or tissue and the concrete metabolic situation.

For example, for the isolated synaptic brain mitochondria, which constitute more than 90% of the total brain mitochondria [27], the optimal substrate mixture is pyruvate + glutamate (neuromediator) [28]. Pyruvate originates from lactate, which in vivo serves as a main substrate for the synaptic mitochondria [29]. Lactate circulates in the blood at mM concentrations [30]. It was generally accepted that most of the lactate originates from glucose. In the brain, liver, and kidney, fatty acid β-oxidation supplies energy and carbon atoms for the increased gluconeogenesis by increasing the formation of OAA [31,32,33]. Both glucose and lactate can be formed from other sugars, such as fructose, galactose, and mannose.

The tricarboxylic acid cycle is usually considered a sequence of biochemical reactions aimed at the oxidation of the main metabolites. The rate of individual reactions may vary, but it was generally accepted that the TCA cycle almost always begins with the synthesis of citrate [34]. The first metabolite of the TCA cycle citrate is formed by condensation of oxaloacetate (OAA) with acetyl-CoA. The latter is produced either by decarboxylation of pyruvate, in the case of glycolysis, or by β-oxidatation of fatty acids. Then, the TCA cycle continues clockwise until the enzyme system returns to the formation of a new citrate molecule from the resulting OAA and a new molecule of acetyl-CoA. During the cycle, 2 molecules of CO_2_, 3 molecules of NADH, and 1 molecule of FADH_2_ are released. However, in vivo, such a scenario could be one of several.

**Figure 2:**
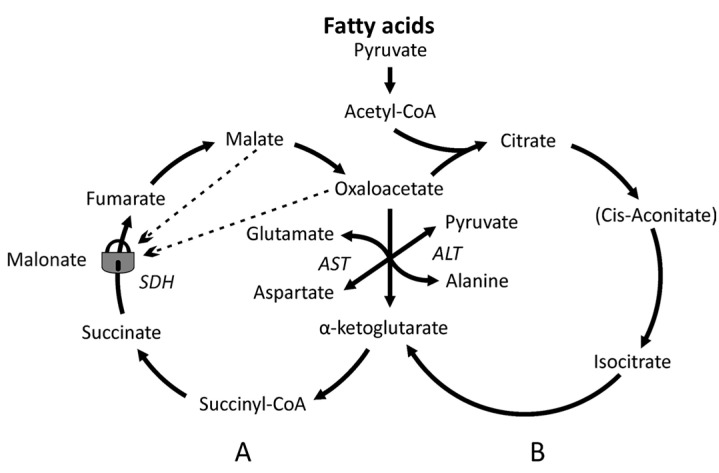
Schematic presentation of the TCA cycle working in the respiring mitochondria in the presence of malate-aspartate shuttle. Abbreviations: AST—aspartate aminotransferase, ALT—alanine aminotransferase, and SDH—succinate dehydrogenase. The red lock indicates the place of inhibition of succinate dehydrogenase activity by malonate and OAA. The dotted red lines from malate and oxaloacetate mean that they are also succinate dehydrogenase inhibitors. The figure was reprinted from [28].

Back in the early 90s, it was shown that in the brain synaptic mitochondria the slowest step of the TCA cycle is between OAA and citrate [34]. In the isolated synaptosomes the activity of citrate synthase was found to be very high, whereas the activity of the pyruvate dehydrogenase complex (PDHC) was 10 times lower [35]. The flux between α-ketoglutarate (α-KG) and OAA was 3–5 times faster, depending on the presence or absence of glucose [34]. This suggests that in the brain, the TCA cycle may function as two conjugated cycles (Figure 2). The inhibition of PDHC becomes understandable in the light of recent evidence that in vivo glycolysis always leads to the formation of lactate, not pyruvate [25,36,37]. Therefore, in vivo, the glycolytic pyruvate will almost never be a source of citrate for the initiation of the TCA cycle. Most mitochondria possess the lactate dehydrogenase that provides pyruvate + H^+^ for oxidation [21,38]. Therefore, lactate serves as a transport form of pyruvate.

In the presence of glutamate, pyruvate and malate, the tricarboxylic acid cycle can function as two conjugated cycles (Figure 2) [34]. Cycle A metabolizes from α-ketoglutarate to OAA, and cycle B metabolizes from OAA to α-ketoglutarate. The flow of substrates through cycle A can be 3–5 times greater than through cycle B. For the brain and spinal cord mitochondria, a mixture of glutamate + pyruvate + malate has been shown to be the optimal substrate mixture [21]. 

Until recently, this phenomenon was considered a case of synaptic metabolism and had not been investigated. However, taking into consideration that glycolysis always ends with the formation of lactate, and that lactate is an important substrate for the mitochondria of most active organs, it makes sense to explain in more detail the physiological meaning of the TCA cycle functioning as two conjugated cycles. The following functions of the two TCA cycles can be distinguished.

**Figure 3:**
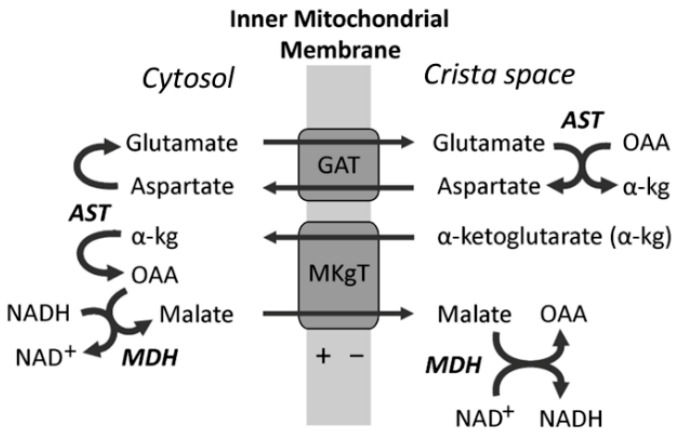
Malate-aspartate shunt (MAS). The inner mitochondrial membrane is impermeable to NADH. In order to effectively use lactic acid as a substrate for respiration, lactate must first be converted to pyruvate in the reaction Lactate + NAD^+^ → Pyruvate + NADH + H^+^. In energized mitochondria, MAS causes lactate dehydrogenase to work irreversibly towards proton oxidation. The driving force for this is the electrogenic exchange of aspartate for glutamate. Abbreviations: AST—aspartate aminotransferase; α-kg—α-ketoglutarate; GAT—glutamate/aspartate transporter; MKgT—malate/ketoglutarate transporter; MDH—malate dehydrogenase; OAA—oxaloacetate. The figure was reprinted from [33].

1. On the cytoplasmic side of the mitochondria, LDH converts lactate into pyruvate and H+ to form NADH. For fast oxidation of pyruvate, it is necessary to rapidly oxidize the cytoplasmic NADH and transfer the electrons into the space of the cristae. For this purpose, there is a malate aspartate shunt (MAS) (Figure 3). MAS transports electrons by transferring reducing equivalents of NADH from the cytoplasm to the crista space. MAS consists of two enzymes, malate dehydrogenase (MDH) and aspartate aminotransferase (AST), located on either side of the cristae membrane (Figure 3), and two carriers across the cristae membrane: glutamate/aspartate transporter (GAT) and malate/α-ketoglutarate transporter (MKgT). The importance of MAS has been proven in experiments with inhibitors of the transaminase and SDH [39]. Although aminotransferases are reversible enzymes, in MAS, AST works only in one direction: in the cytoplasm, aspartate + α-ketoglutarate → OAA + glutamate, and in the cristae, OAA + glutamate → aspartate + α-ketoglutarate (Figure 3).
2. In the presence of pyruvate and glutamate in the same space, alanine aminotransferase catalyzes the reaction: Pyruvate + Glutamate → Alanine + α-ketoglutarate. Since glutamate is constantly entering the mitochondria during the work of MAS, and α-ketoglutarate is constantly removed from the cristae space and oxidized in the cycle A (Figure 2), ALT works only towards the formation of alanine. The physiological meaning of this process is that in most organs, except the liver, the mitochondria lack the enzyme glutamate dehydrogenase. Therefore, toxic ammonium released during glutamate oxidation, especially in the brain, is captured during the formation of alanine by ALT. Alanine from the muscles and brain enters the bloodstream and travels to the liver, where ALT works backwards to form pyruvate and glutamate. Pyruvate in the liver is a substrate for active gluconeogenesis, especially in the glucose–alanine cycle: alanine → pyruvate (carboxylation) → OAA → phosphoenolpyruvate → … → glucose. Therefore, lactate is the transport form of pyruvate for oxidation by mitochondria, and alanine is the transport form of pyruvate for gluconeogenesis. Alanine is also the source of ammonium for the formation of urea in the liver. Since the direction of the reactions of AST and ALT can change in different compartments or organs, in the general scheme of Figure 3, arrows point in both directions.
3. The excess of α-ketoglutarate, formed by ALT and AST, rapidly undergoes transformations in cycle A, creating an excess of OAA, which in turn becomes a substrate for cycle B. Both cycles become conjugated and are irreversible. This allows intermediate metabolites of cycle B to be directed into other metabolic pathways. Depending on the organ, citrate can be directed to lipid synthesis, accumulate in prostatic fluid, or be converted to isocitrate and used in the transhydrogenase reactions to transfer the mitochondrial redox equivalents of NADH to the cytosolic NADPH [40].

**Figure 4:**
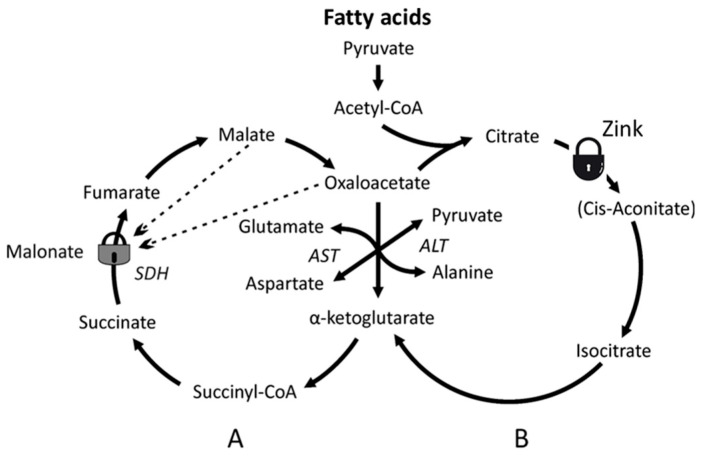
Functioning of the TCA cycle in the prostate mitochondria. Inhibition of aconitase by zinc ions leads to the accumulation of citrate and inhibition of the minor B cycle. The figure was reprinted from [41].

Another very interesting example of the functional adaptation of the TCA cycle to the needs of an organ are mitochondria of the prostate. In the prostate of sexually mature men, mitochondria produce huge amounts of citrate. This has been found to be due to the inhibition of aconitase by zinc ions [40]. Therefore, the TCA cycle of the prostate mitochondria cannot work according to the classical scheme. Acetyl-CoA condenses with oxaloacetate to citrate, which is excreted entirely into a prostatic liquid, where the concentration of citrate can reach 10 mM. Under these conditions, only the small cycle A can fully function in the mitochondria of the prostate (Figure 4), where amino acids are used as a source of α-ketoglutarate. That is probably why men need to eat meat, which stimulates the prostate.

The physiological meaning of the high citrate concentration in the prostatic fluid is that Ca^2+^ ions are necessary for the active work of the spermatozoid’s tail. Therefore, when prostatic fluid mixes with spermatozoa, the citrate chelates Ca^2+^ and thus immobilizes spermatozoids. At the right time and in the right place, calcium is released, the spermatozoids begin wagging their tails, and if one of them is successful, the citrate will serve as a substrate for the zygote.

## 3. Respiratory Functions of the Isolated Mitochondria

From the discussion of the metabolic properties of mitochondria we excluded liver mitochondria. This is because the liver and liver mitochondria have a number of exceptional functions and properties that other organs and their mitochondria do not have. For example, the liver and liver mitochondria are relatively resistant to prolonged hypoxia and are able to endure complete ischemia within 20–30 min. The properties of the liver mitochondria vary greatly, depending on the composition of the food and the time that has passed after the last meal. Liver mitochondria have a large osmotically active volume and have a large storage of endogenous substrates; the liver is capable of active regeneration, etc. [42].

**Figure 5:**
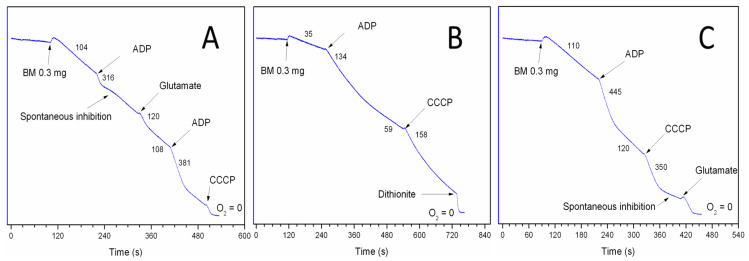
Oxygen consumption by rat heart mitochondria, isolated without BSA, oxidizing succinate, palmitoyl-carnitine, and their mixture in different metabolic states. (**A**) Succinate 5 mM; (**B**) palmitoyl-carnitine 25 µM; and (**C**) palmitoyyl-carnitine + succinate.

As regards mitochondria from other than liver organs, the most difficult obstacles have been met during attempts to study respiratory activities with seemingly “simple” physiological substrates, namely succinate and fatty acyl carnitines. Figure 5 illustrates an experiment with the isolated synaptic mitochondria from the Sprague–Dawley rat. We mention the strain of animals because many functions, including respiratory activities, are species-specific. This is important when comparing data from different species and strains of the same species. The composition of incubation medium, substrate concentrations, and additions are described in Figure 5. For the readers not familiar with the polarographic assay, we give some explanation. Changes in O_2_ concentration in the incubation medium were measured with the platinum Clark electrode [42]. The rates of O_2_ consumption are in nanomoles of O_2_ consumed per one minute per mg of mitochondrial protein. Substrates were added before mitochondria. Metabolic states are as follows: State 4, or resting respiration—O_2_ consumption before the addition of ADP; State 3, or active oxidative phosphorylation—the respiration rate after addition of ADP; and uncoupled respiration—oxygen consumption after the addition of a protonophore (CCCP), which collapses (uncouples) the membrane potential. 

It should be stressed that endogenous inhibitions of SDH in mitochondria are highly variable and species-specific. Some species display total inhibition of SDH [43]. Figure 5A shows that addition of glutamate totally released the inhibition of SDH.

Figure 5A shows oxidation of succinate by rat heart mitochondria. Figure 5B shows oxidation of palmitoyl-carnitine. The patterns shown in Figure 5A,B are also typical for the brain and kidney mitochondria [44]. The rates of O_2_ consumption during P-C oxidation strongly depend on the time passed after the isolation of mitochondria. This indicates that the endogenous substrates might have a stimulatory effect. Indeed, Figure 5C shows that in the presence of succinate the rates of oxidative phosphorylation are the highest. The increased State 4 respiratory rates with succinate plus P-C were caused not by the uncoupling, but by the increased reverse electron transport associated with the increased content of the membrane’s ubiquinol. Both succinate and P-C reduce ubiquinone to ubiquinol [8].

Incubation medium: 125 mM KCl, 10 mM NaCl, 10 mM MOPS, pH 7.2, 2 mM MgCl_2_, 2 mM KH_2_PO_4_, 1 mM EGTA, and 0.7 mM CaCl_2_. At Ca^2+^/EGTA = 0.7, the [Ca^2+^]_Free_ was 1 µM. Chamber volume = 0.65 mL. Substrates: succinate, 5 mM; palmitoyl carnitine, 25 µM. Numbers in the traces are respiratory activities in nmol/min/mg mitochondrial protein. Additions: rat heart mitochondria 0.3 mg, ADP 150 µM, CCCP 0.5 µM, and glutamate 5 mM.

High rates of oxidative phosphorylation with palmitoyl-carnitine + succinate were also observed with the kidney mitochondria [45] and the brain [33]. Because kidney mitochondria do not have endogenous inhibition of SDH, they oxidize succinate at a very high rate. Respiratory rate in State 3 with P-C + succinate was only 30% higher than with succinate alone. So, we did a control experiment with 0.5 mM succinate. There was no oxygen consumption by the kidney mitochondria with 0.5 mM succinate alone; however, 0.5 mM succinate stimulated P-C oxidation 4-fold and 8-fold the oxidation of octanoyl-carnitine [45].

**Figure 6:**
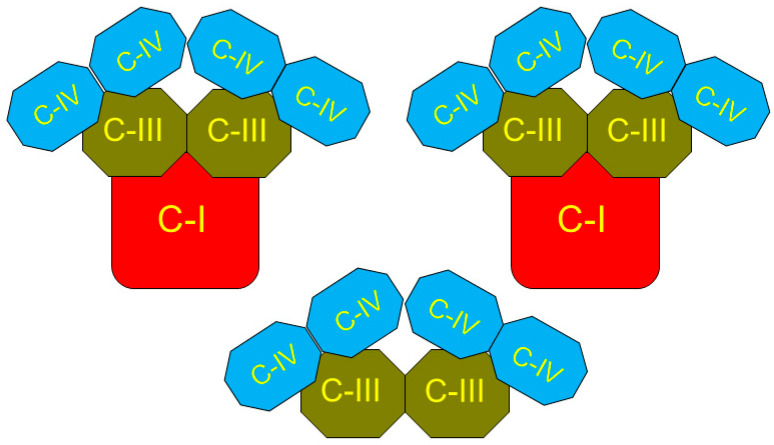
Schematic structure of the respirasome as suggested by Schägger [7]. The figure depicts two large and one small supercomplexes of the respiratory chain carriers. They are integral structures that span the inner membrane and the figure shows the view from the matrix side. The figure was reprinted from [46].

It was not possible for us to explain the above facts from the point of view of the old paradigms. We tried to interpret the data of Figure 5 from the standpoint of the respirasome as a functional unit for respiration, as shown in Figure 6.

## 4. Respirasome

### 4.1. History

In the 1980s, it was shown that for the beef heart mitochondria the single set of respiratory complexes, involved in oxidative phosphorylation, namely complexes I:II:III:IV:V, were related as 1:2:3:6–7:3–5 [47]. In 2000, Schägger and Pfeiffer (2000) presented evidence that the electron carriers are organized into three supercomplexes [6]. By interacting together, the three supercomplexes form a functional superstructure named “respirasome” [6]. Sometime after the discovery of respirasome, researchers started to designate the stoichiometry of complexes as I_1_III_2_IV_4_ [48] and found a significant variability in composition of the respirasomes among different species [49,50]. The respirasome presented by Schägger (2001) contained two large supercomplexes and one smaller supercomplex [7]. Each of the large supercomplexes comprised a monomer of complex I, a dimer of complex III, and two dimers (four copies) of complex IV (Figure 6). The smaller supercomplex consisted of one dimer of complex III and two dimers of complex IV, or III_2_ IV_4_ [7]. The two active centers in the dimer of complexes III in the smaller supercomplex open into the lipid phase of the inner membrane and react directly with the membrane’s pool of ubiquinol. In the large supercomplexes the active centers of complexes III do not interact with the membrane’s pool of coenzyme-Q. Sousa et al. (2016) have shown that in the large supercomplex, only one of the two Rieske iron–sulfur domains of the complex III dimer was active, indicating that the other monomer of complex III dimer was inactive [51].

The redox potential difference (ΔE°′) between ubiquinol (QH_2_) (+0.045 V), which donates electrons to Complex III, and O_2_/H_2_O (+0.82 V), the terminal acceptor at Complex IV, is about 0.77 V. The energy released by the work of the small supercomplex (CIII_2_CIV_2_) is very high: ∆G = −nF∆E = −148 kJ per mol [52]. Therefore, the rate of ubiquinol oxidation is very fast, irreversible, and releases a lot of heat.

It is evident that the membrane’s pool of the reduced Co-QH_2_, which swims inside the liquid lipid phase of the inner membrane at the temperature of +50 °C [53], can be oxidized only by the smaller supercomplex of the respirasome. This reaction is the main source of the irreversibility in the cell.

### 4.2. Respirasome Structure

After the discovery of the respirasome structure [4,6], a rather large number of papers describing various aspects of supercomplexes organization were published, of which we mention a few [48,53,54,55,56,57,58]. The respirasome presented by Schägger (2001) contained two large supercomplexes and one smaller supercomplex [7] (Figure 6).

**Figure 7:**
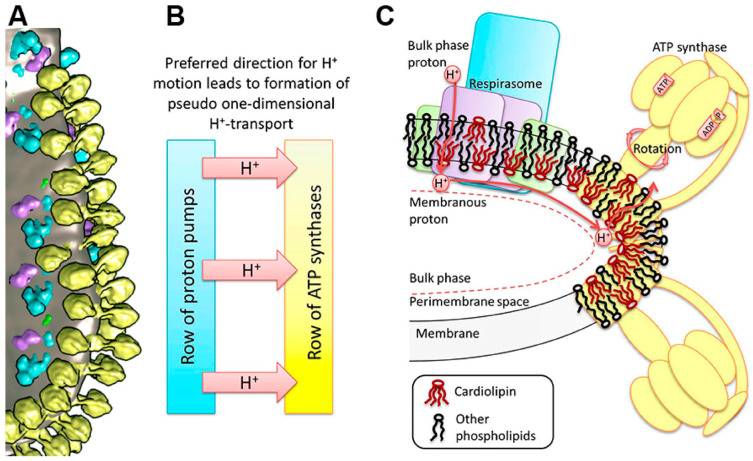
Structure of the mitochondrial OXPHOS system and cristae membrane illustrating a proton transfer pathway. (**A**) The cluster of components of the OXPHOS system at the bend of crista of heart mitochondria [55]. Yellow—ATP synthase dimers, blue—complex I, purple—complex III dimers, green—complex IV, and gray—lipid membrane. (**B**) A dedicated direction of proton transfer between rows of proton pumps and ATP synthases. (**C**) Schematic reconstruction of the cluster in the OXPHOS system on the membrane fold and a pathway of the lateral transfer of protons from the respirasome to ATP synthase. The area of increased curvature of the membrane is enriched with CL molecules. The figure was reprinted from [11]. The blue color in Figure 7 depicts structures related to the electron transport; yellow color depicts structures related to ATP synthase.

Some authors observed that attenuation of the biogenesis of individual respiratory chain complexes was accompanied by increased formation of stable respiratory supercomplexes. This phenomenon was not accompanied by increased mitochondrial respiratory activity. Therefore, it was concluded that formation of the supercomplexes is necessary for the structural stabilization, but not for the enhancement of the respiratory chain catalysis [59]. Some authors believe that the term “respirasome” describes the phenomenon of formation in some organisms of the respiratory complexes’ clusters, but the complexes themselves can work independently [60]. It was shown that clusters of the respiratory complexes form the “respiratory string” [9,10,11,12], as shown in Figure 7A,B. 

Association of basic units into a string is mediated by complex IV, which interacts with the neighboring complex IV through a dimeric interface found in the X-ray structure [9]. The basic unit of the respiratory string has the structure I_2_ III_2_ IV_2_, thus indicating that the respirasome may be a structure of the higher order. The association of the base units into a chain is provided by complex IV, which forms dimers with complex IV of the adjacent basic unit. Some authors presented different ratios and compositions of supercomplexes in the respirasomes [51,61].

## 5. The Key Roles of Fatty Acids β-Oxidation and Lactate Accumulation and Oxidation in Human Metabolism

### 5.1. Fatty Acids β-Oxidation

**Figure 8:**
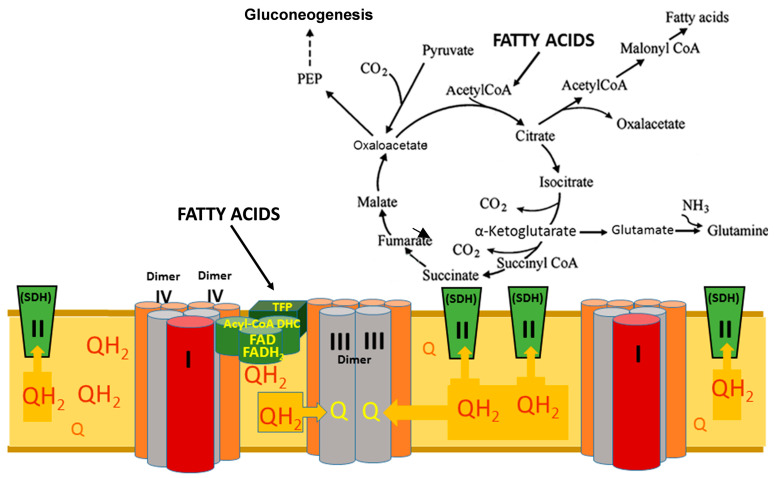
Functioning of the respirasome and the tricarboxylic acid cycle during active β-oxidation of long-chain fatty acids. Abbreviations: Acyl-CoA DHC—acyl-CoA dehydrogenase complex, which includes three enzymes: acyl-CoA dehydrogenase, electron transfer flavoprotein (ETF), electron-transferring-flavoprotein dehydrogenase (ETFDH); PEP—phosphoenolpyruvate; TFP—trifunctional protein of the β-oxidation of fatty acids system; SDH—succinate dehydrogenase; Q—ubiquinone, oxidized form of coenzyme Q; and QH_2_—ubiquinol, reduced form of coenzyme Q. The figure reprinted from [45].

The highest rates of ubiquinone reduction to ubiquinol occur in the organs where β-oxidation of long-chain fatty acids is an important source of ATP and NADPH. Accordingly, the highest steady-state levels of CoQH_2_ are maintained in these organs, high enough to initiate the reversal of the electron flow from CoQH_2_ to the TCA cycle [15]. This increases the level of NADH in the mitochondria and the presence of the energy-dependent transhydrogenase translates a significant part of the mitochondrial redox potential NADH/NAD+ to the cytosolic NADPH/NADP+ [62,63]. Simultaneous increases in ATP and NADPH production stimulate anabolic and anaplerotic metabolic pathways as well as the main physiological functions of the organs (Figure 8).

The existing fallacy that mitochondrial respiration and energization depend on the external redox potential is correct only for the in vitro conditions when the isolated mitochondria lose metabolic communication with the cell and blood circulation. In the well-energized isolated mitochondria, the reverse electron transfer, which occurs during oxidation of the FAD-dependent substrates, restores the components of complex I, leading to increased production of superoxide radicals [15,64,65]. Under the in vitro experimental conditions, the excess of electrons cannot be directed to the cytoplasm because there are no conditions for activation of the mitochondrial transhydrogenase (MTH). It can be expected that in vivo there is no stimulation of the formation of free radicals, at least not to the same extent as in vitro, since MTH and the reversal of the transfer of electrons through SDH (complex II) dump the excess of energy and electrons into the cytoplasm.

The high level of the redox potentials in the cytoplasm leads to the fact that glycolysis always results in the formation of lactate, rather than pyruvate [22,25,36]. Since pyruvate and other ketoacids are very unstable, we can consider lactate as the storage and transport form of pyruvate and the redox buffer. Most mitochondria have active lactate dehydrogenase that produces free pyruvate and a proton, which are oxidized by the mitochondria [66,67,68].

### 5.2. Oxidation of Lactate and Fatty Acids During Exercise

In this section, we discuss only the energy functions of lipids and carbohydrates, leaving aside numerous regulatory functions, such as acetylation, glycosylation, lactosylation, or the formation of prostaglandins, etc. The functional energy requirements of different organs vary significantly. For example, the kidneys are constantly working and use only fatty acids as the energy source as glucose is used to reabsorb sodium. In addition, due to active gluconeogenesis, the kidneys, together with the liver, maintain blood glucose homeostasis and produce lactate. Other organs may change their functional load, sometimes many times. For example, skeletal muscle can increase oxygen consumption by 40-fold and the heart by 8–9-fold [69]. The rates of oxidative phosphorylation by the isolated mitochondria from any organs of mice and rats are approximately the same and are limited by the rate of ATP synthase [13]. Even if the rate of ATP production by mitochondria in vivo would be several times higher than in vitro, mitochondria cannot provide enough ATP if, for example, the heart increases its energy demand by 9 times during intensive physical activity. Obviously, there must be an additional source of energy.

The discovery of the lactate cycle and the forced formation of lactate instead of pyruvate is a key event in the history of bioenergetics [21,22,36,37]. It becomes evident that lactate plays important roles in the overall energy balance of the human body [13,22]. A good confirmation of this thesis is the data obtained during the studies of metabolic processes in athletes under various physical loads. The main advantage of these studies is that they are carried out on the whole body, although by indirect methods [30,70].

In untrained people, the increase in physical activity leads to increased lactate content and a decrease in the consumption of fatty acids. In professional athletes, the lactate content is kept at a low level for a long time, which indicates a high rate of lactate consumption from the blood. At the same time, the consumption of fatty acids begins to increase. In athletes, the content of blood lactate begins to increase only with heavy loads, when the consumption of fatty acids begins to decrease. These experiments revealed the following important properties of energy metabolism: (1) The rate of consumption of fatty acids is the inverse of the rate of consumption of lactate. This switching from one substrate to another is called “metabolic flexibility.” (2) High adaptability of energy metabolism. Lactate and FA intake can increase greatly with regular exercise. (3) With very intense physical activity, energy metabolism increasingly switches to lactate consumption [30,70].

In spite of very interesting and inspiring data we just discussed, we will not rush to make and globalize the conclusions. Firstly, these results relate primarily to muscle tissue; secondly, these are the results of indirect methods of studying metabolism; and thirdly, we know very little about mitochondrial oxidation of fatty acids and its regulation. Finally, we know too little about the roles of SDH in β-oxidation and the origins of lactate. We still know very little about the origin of the gender differences in energy metabolism. It is especially important to know gender differences in lipid metabolism [71]. Females of all mammals, including humans, live longer than males, produce fewer superoxide radicals, and age more slowly [72,73]. Women oxidize fatty acids differently during exercise, produce fewer superoxide radicals, and age more slowly [reviewed in [74].

## 6. Discussion

In mammals, there are two main sources of biological energy: carbohydrates and fatty acids. There are very large differences in the size of the storage and the rate of metabolism between these two energy sources. Fats have large storage; the metabolic pathways of catabolism and formation of triglycerides and phospholipids are very complex and require numerous cofactors (CoA, carnitine, CoQ, and biotin). In comparison, the storage of glucose, glycogen, and lactate is limited. For these reasons, the two energy sources have different strategic roles in the body.

We suggest that fatty acids serve as a basic source of energy (ATP, NADPH, and gradients), supporting anabolic and anaplerotic functions. Catabolism of fatty acids via the minor supercomplex of the respirasome and complex II maintains, in the body, high levels of reduction in the NAD and NADP systems, supporting the lactate cycle by obligatory reduction in pyruvate to lactate and supporting gluconeogenesis.

Carbohydrates, namely glucose and lactate, have shorter and less complex metabolic steps. Therefore, they have much faster metabolic turnover and more urgent functions in comparison with the fatty acids. Here, we consider only energetic functions. Glucose via glycolysis supplies ATP to the cell’s compartments, which lack mitochondria. For example, in axons of the CNS, lactate accumulates in the blood and can quickly supply pyruvate for oxidation to the organs in the body, which increases their functional activity.

Regretfully, the metabolism of carbohydrates and lipids has been studied independently of each other and mostly in the in vitro system. The main difference between the in vitro system and the whole living organism is that in vitro the connections between various metabolic processes and organs are lost. A striking example of the difference between the in vitro and in vivo metabolic pathways is the fact that in the whole organism, glycolysis always ends in the formation of lactate, not pyruvate [22,30]. Many researchers studied carbohydrates and lactate metabolism without mentioning the existence of lipid metabolism [1,2,3]. As we show in this review, the relationships between β-oxidation of fatty acids determine the obligatory formation of lactate. In its turn, oxidation of lactate strongly affects the TCA cycle and thus influences all types of metabolic events.

## 7. Conclusions and Future Perspectives

The numerous discoveries that have been made during the last several decades force us to reconsider the energy metabolism of mammals and, most importantly, humans. We must discard old notions, such as aerobic-anaerobic glycolysis, and the predominant role of mitochondrial oxidative phosphorylation in the body’s energy supply. Instead of pitting glycolysis and OXPHOS against each other, we must investigate their relationships.

We need to be more careful in transferring the conclusions made in the in vitro experiments to the whole organism and investigate metabolic problems using different approaches. Unfortunately, we still have very little information about the mechanisms of β-oxidation of fatty acids with different aliphatic chain lengths and their physiological roles. We know very little about the key element in the β-oxidation and reverse electron flow: complex II (SDH). Gender differences in metabolism are one of the most pressing problems for biochemistry, physiology, and medicine. Preliminary evidence suggests that these studies may have a huge impact on the understanding of physiology and pathophysiology of metabolic processes in the human body.

And that is not all. In this review, we have touched on only a small part of the new discoveries. There are still many analytical problems ahead that arise with the emergence of the new data. For example, how will the discovery of the fact that mitochondrial cristae are independent cellular compartments affect our understanding of the mechanisms of OXPHOS and energy metabolism in general?

## Data Availability

No new data were created or analyzed in this study.
